# Single-agent activity of phosphatidylinositol 3-kinase inhibition with copanlisib in patients with molecularly defined relapsed or refractory diffuse large B-cell lymphoma

**DOI:** 10.1038/s41375-020-0743-y

**Published:** 2020-02-14

**Authors:** Georg Lenz, Eliza Hawkes, Gregor Verhoef, Corinne Haioun, Soon Thye Lim, Dae Seog Heo, Kirit Ardeshna, Geoffrey Chong, Jacob Haaber, Wei Shi, Igor Gorbatchevsky, Susanne Lippert, Florian Hiemeyer, Paolo Piraino, Georg Beckmann, Carol Peña, Viktoriya Buvaylo, Barrett H. Childs, Gilles Salles

**Affiliations:** 1 Department of Medicine A, Hematology, Oncology, and Pneumology, University Hospital Münster, Münster, Germany; 2 Eastern Health Clinical School, Monash University, Olivia Newton John Cancer Research and Wellness Centre, Melbourne, VIC Australia; 3 University Hospitals Leuven, Leuven, Belgium; 4 Lymphoid Malignancies Unit, Groupe Hospitalier Henri Mondor-Albert Chenevier, Creteil, France; 5 National Cancer Centre Singapore and Duke-NUS Medical School, Singapore, Singapore; 6 Department of Internal Medicine, Seoul National University Hospital, Seoul, South Korea; 7 University College London Hospitals NHS Foundation Trust, London, UK; 8 Ballarat Regional Integrated Cancer Centre, Ballarat, VIC Australia; 9 Department of Hematology, Odense University Hospital, Odense, Denmark; 10 Bayer China, Beijing, China; 11 Bayer HealthCare Pharmaceuticals, Inc., Whippany, NJ USA; 12 Pharmaceuticals Division, Bayer AG, Berlin, Germany; 13 Hospices Civils de Lyon, Université de Lyon, Centre Hospitalier Lyon-Sud, Service d’hématologie, Lyon, France

**Keywords:** B-cell lymphoma, Targeted therapies

## Abstract

Patients with relapsed/refractory diffuse large B-cell lymphoma (DLBCL) have adverse outcomes. We evaluated the efficacy and safety of the phosphatidylinositol 3-kinase inhibitor copanlisib in patients with relapsed/refractory DLBCL and assessed the relationship between efficacy and DLBCL cell of origin (COO; activated B-cell like [ABC] and germinal center B-cell like [GCB]) and other biomarkers. The primary endpoint was objective response rate (ORR) in DLBCL COO subgroups (ABC, GCB, and unclassifiable) and by *CD79B* mutational status (NCT02391116). Sixty-seven patients received copanlisib (ABC DLBCL, *n* = 19; GCB DLBCL, *n* = 30; unclassifiable, *n* = 3; missing, *n* = 15). The ORR was 19.4%; 31.6% and 13.3% in ABC and GCB DLBCL patients, respectively. ORR was 22.2%/20.0% for patients with/without *CD79B* mutations (wild type, *n* = 45; mutant, *n* = 9; missing, *n* = 13). Overall median progression-free survival and duration of response were 1.8 and 4.3 months, respectively. Adverse events included hypertension (40.3%), diarrhea (37.3%), and hyperglycemia (32.8%). Aberrations were detected in 338 genes, including *BCL2* (53.7%) and *MLL2* (53.7%). A 16-gene signature separating responders from nonresponders was identified. Copanlisib treatment demonstrated a manageable safety profile in patients with relapsed/refractory DLBCL and a numerically higher response rate in ABC vs. GCB DLBCL patients.

## Introduction

Malignant lymphoma encompasses a heterogeneous group of malignancies [1]. Diffuse large B-cell lymphoma (DLBCL), characterized by aggressive clinical behavior, is the most frequent subtype, accounting for ~30–40% of new cases diagnosed globally [2].

While most patients with DLBCL achieve a durable response to standard-of-care first-line chemotherapy of rituximab with cyclophosphamide, doxorubicin, vincristine, and prednisone (CHOP) [3, 4], approximately one-third of patients develop disease that is refractory to, or has relapsed after, initial response [5]. Patients with relapsed/refractory DLBCL are characterized by adverse prognosis [6, 7]. High-dose chemotherapy followed by autologous stem cell transplantation remains the current standard of care for patients with relapsed/refractory DLBCL, although long-term outcomes are poor [7]. In addition, some patients are ineligible due to age or contraindicating comorbidities [5, 8]. Novel therapies are urgently required to improve outcomes for DLBCL patients.

Gene-expression profiling distinguishes at least two major molecular DLBCL subtypes, reflecting the cell of origin (COO): activated B-cell-like (ABC) DLBCL and germinal center B-cell-like (GCB) DLBCL [1, 9]. These subtypes differ in their gene-expression profiles, clinical outcomes, prognosis, and responsiveness to targeted therapies [10, 11]. ABC DLBCLs frequently harbor *CD79B* mutations, resulting in chronic active B-cell receptor (BCR) signaling, an important pathogenetic mechanism in these lymphomas [12]. In preclinical studies, targeted inhibition of the downstream BCR-signaling molecules phosphatidylinositol 3-kinase (PI3K)-α and -δ has demonstrated significant antilymphoma activity, particularly in models of ABC DLBCL [13–15]. In addition, in vitro and in vivo data have suggested that ABC DLBCL models bearing *CD79B* and/or *MYD88* mutations may be more sensitive to inhibition of PI3K-α and -δ isoforms than other molecular DLBCL subtypes [14]. Whereas data from a small phase I study indicated that selective inhibition of PI3K-δ was ineffective in treating DLBCL [16], subsequent preclinical studies demonstrated that dual inhibition of PI3K-α and -δ isoforms may be necessary for effective treatment of DLBCL [14, 15, 17]. Indeed, inhibition of PI3K-δ alone may be self-limiting as it has been shown to result in feedback activation of PI3K-α and BCR signaling in ABC DLBCL cell lines [17].

Copanlisib (Aliqopa; Bayer AG, Berlin, Germany) is a pan-class I PI3K inhibitor with potent activity against PI3K-α and -δ isoforms [18]. A first-in-human phase I study determined the maximum tolerated dose of copanlisib to be 0.8 mg/kg administered on days 1, 8, and 15 of a 28-day cycle, with an expansion cohort demonstrating satisfactory safety and a partial response (PR) in a patient with unselected DLBCL (objective response rate [ORR] 1/3 patients; 33%) on treatment more than 16 months [19]. A phase II study in 48 patients with aggressive lymphoma, including 15 patients with unselected DLBCL, observed an overall ORR of 27% with one PR in a DLBCL patient [20]. Lastly, a recent phase II study of copanlisib in pretreated patients with relapsed/refractory indolent lymphoma demonstrated promising efficacy (ORR 84/142 patients; 59%) and manageable toxicity [21]. Based on these results, we conducted a phase II study evaluating the efficacy and safety of copanlisib in patients with relapsed/refractory DLBCL. We also evaluated the molecular profiles of patients to potentially identify subgroups that may benefit most from copanlisib by assessing the relationships between efficacy and these potential candidate predictive biomarkers. We believe this is the largest study to date investigating a PI3K inhibitor in patients with relapsed or refractory DLBCL.

## Materials and methods

### Study design and participants

This single-arm, open-label, multicenter, phase II study (NCT02391116) evaluated the efficacy and safety of single-agent copanlisib in patients with relapsed/refractory DLBCL. Patients eligible for inclusion were aged ≥18 years, with a histologically confirmed diagnosis of de novo DLBCL, or DLBCL transformed from follicular lymphoma (FL) according to the World Health Organization classification [22]. Eligible patients had received one or more lines of prior therapy for DLBCL, including rituximab with CHOP or CHOP-like regimens, and were not eligible or not willing to receive high-dose myeloablative chemotherapy and stem cell transplantation. Additional eligibility and exclusion criteria are included in the Supplementary information.

The study was compliant with the Declaration of Helsinki and Good Clinical Practice and was approved by the appropriate ethics committees prior to the start of recruitment. All participants provided written, informed consent before study entry.

### Copanlisib treatment

Patients received copanlisib 60 mg as a 1 h intravenous infusion on days 1, 8, and 15 of a 28-day cycle. Dose reductions to 45 mg were permitted in the event of toxicities. Treatment continued until disease progression or unacceptable toxicity. All patients completed a 30-day safety follow-up after the last dose except for patients lost to follow-up or who had died. Patients who discontinued treatment for any reason other than radiologic disease progression entered active follow-up, which encompassed the safety follow-up.

### Objectives and assessments

The primary objective was to assess the efficacy of copanlisib in patients with relapsed/refractory DLBCL and to evaluate the relationship between efficacy and potentially predictive biomarkers, specifically DLBCL COO and *CD79B* mutational status. Secondary objectives were to assess other radiologic and survival indicators of treatment efficacy, safety, and tolerability of copanlisib, and long-term effects of treatment.

The primary efficacy variable was ORR, defined as the proportion of patients who had an overall response of complete response (CR) or PR according to Lugano 2014 criteria [23], based on the investigator’s assessment; these patients were considered to have shown an objective response and were deemed responders. Response was assessed in biomarker subgroups based on DLBCL COO (ABC, GCB, or unclassifiable) and *CD79B* mutational status. All other biomarker analyses were exploratory.

Secondary efficacy variables included duration of response (DoR), progression-free survival (PFS), overall survival, duration of stable disease, and disease control rate (DCR); these variables are defined in the Supplementary information.

Radiologic tumor assessments by computed tomography/magnetic resonance imaging scan or positron emission tomography–computed tomography scan were performed at baseline, every 8 weeks during treatment, and every 12 weeks during active follow-up. Safety was assessed continually throughout the study through clinical laboratory variables, physical examinations, Eastern Cooperative Oncology Group performance status, vital signs, 12-lead electrocardiogram, and cardiac function. Adverse events (AEs) were graded using the National Cancer Institute Common Terminology Criteria for Adverse Events version 4.03.

The overall cohort was defined as patients assigned to treatment (full analysis set). The per-protocol set (PPS) was defined as patients from the overall cohort who had a tumor biopsy available at baseline (COO and *CD79B* results), completed at least one treatment cycle (three doses), had no major protocol deviation affecting the primary efficacy evaluation, and had at least one postbaseline tumor assessment (or discontinuation due to death or progression by clinical judgement before tumor assessment). Results of the primary analysis are presented here for the PPS and overall cohort. Safety variables were analyzed using the overall cohort. Additional results are presented for the overall cohort, with additional results for the PPS available in the Supplementary information.

### *CD79B* and DLBCL COO assays and biomarker analysis

Pretreatment tumor biopsy samples were mandatory for biomarker analysis (either fresh samples collected at screening and/or archival formalin-fixed, paraffin-embedded samples collected after the last relapse or disease progression). *CD79B* mutational status and other variants were assayed using next-generation sequencing (NGS) with the FoundationOne Heme panel (Foundation Medicine, Cambridge, MA, USA), which tested for variants in ≥400 genes, including selected rearrangements/fusions frequently reported in hematological malignancies. DLBCL COO was determined using the EdgeSeq COO assay (HTG Molecular Diagnostics, Inc., Tucson, AZ, USA [HTG EDGE-SQ-100]) based on mRNA-expression levels of a 22-gene panel.

### Statistical methods

The primary efficacy analysis of ORR was performed on the overall cohort and PPS for DLBCL COO and *CD79B* biomarkers, with PPS being the primary analysis set as it allowed for precise estimation and comparison of the COO and *CD79B* biomarker effect. Differences of ORR in the respective biomarker-positive group minus ORR in the complementary (biomarker-negative) group were calculated. Two-sided 90% confidence intervals (CIs) for the differences were provided. *P* values for exploratory purpose were calculated using Barnard’s unconditional exact test. ORRs for the PPS and overall cohort were also calculated. For the ORR, exact two-sided 90% CIs based on Clopper–Pearson methodology were provided. For secondary efficacy variables, the overall cohort was used for the main analysis. DoR, PFS, overall survival, and duration of stable disease were presented using the Kaplan–Meier method, and medians with 95% CIs were calculated. The study was conceptualized as an estimation study and sample size was chosen based on expected length of the CI for ORR difference. Additional methods for sample size planning and statistical power can be found in the Supplementary information.

Additional mutational status (excluding those for the primary efficacy analysis) was evaluated for correlation with clinical endpoints, using Fisher’s exact test for response and disease control endpoints, and Kaplan–Meier plots as well as hazard ratios determined by Cox regression models for PFS.

Exploratory analysis of mutation-based signatures was performed. A subset of 134 genes was analyzed based on the presence of at least three patients with mutations in these genes. Correspondence discriminant analysis [24] was used to separate samples on the basis of investigator-assessed treatment response, and a composite score was calculated; additional details are provided in the Supplementary information.

## Results

Sixty-seven patients were assigned to treatment and comprised the overall cohort (Table 1). The median age at baseline was 69 years (range 25–93), 58.2% were male, and the majority of patients (56.7%) had an Eastern Cooperative Oncology Group performance status of 1. Patients had received a median of three (range 1–13) prior lines of antilymphoma therapy, and 52 patients (77.6%) were refractory against their last systemic treatment (Table 1). Two patients (3.0%), one each with ABC DLBCL and GCB DLBCL, had received a prior autologous stem cell transplant. Fourteen patients had DLBCL transformed from FL. Fifty-six patients entered the safety follow-up.

**Table 1:** Patient demographics and baseline characteristics by ABC DLBCL, GCB DLBCL, and in the overall cohort.

	ABC DLBCL *n* = 19	GCB DLBCL *n* = 30	Overall cohort *N* = 67^a^
Age (years), median (range)	72.0 (41–84)	67.0 (32–85)	69.0 (25–93)
Age ≥65 years, *n* (%)	15 (78.9)	17 (56.7)	40 (59.7)
Male, *n* (%)	11 (57.9)	17 (56.7)	39 (58.2)
ECOG performance status score, *n* (%)			
0	5 (26.3)	8 (26.7)	15 (22.4)
1	11 (57.9)	16 (53.3)	38 (56.7)
2	3 (15.8)	6 (20.0)	14 (20.9)
Histology of tumor, *n* (%)			
DLBCL transformed from FL	3 (15.8)	9 (30.0)	14 (20.9)
DLBCL not otherwise specified	16 (84.2)	21 (70.0)	51 (76.1)
EBV-positive DLBCL of the elderly	0	0	1 (1.5)
T-cell/histocyte-rich large B-cell lymphoma	0	0	1 (1.5)
Stage at study entry, *n* (%)			
I	0	3 (10.0)	3 (4.5)
II	0	6 (20.0)	9 (13.4)
III	4 (21.1)	7 (23.3)	11 (16.4)
IV	15 (78.9)	14 (46.7)	44 (65.7)
Median time from initial diagnosis to start of study treatment, months (range)	16.8 (1.4–123)	41.4 (0.7–192)	17.1 (0.7–192)
Median time since first progression, months (range)	3.0 (0.2–60.5)	6.9 (0.7–184)	4.3 (0.2–184)
Median time from most recent progression to start of study treatment, months (range)	0.8 (0.2–3.0)	1.3 (0.2–6.2)	5.0 (0.2–6.2)
Median prior anticancer therapy lines, *n* (range)	2 (1–5)	3.5 (1–13)	3 (1–13)
Median time since last systemic anticancer therapy, months (range)	1.7 (0.7–118)	1.6 (1.0–68.5)	1.9 (0.7–118)
Refractory against last systemic anticancer therapy, *n* (%)			
Yes	15 (78.9)	24 (80.0)	52 (77.6)
No	4 (21.1)	6 (20.0)	15 (22.4)

*ABC* activated B-cell like, *COO* cell of origin, *DLBCL* diffuse large B-cell lymphoma, *EBV* Epstein−Barr virus, *ECOG* Eastern Cooperative Oncology Group, *FL* follicular lymphoma, *GCB* germinal center B-cell like.

^a^Includes three unclassifiable patients and 15 patients with data missing for COO.

**Fig. 1:**
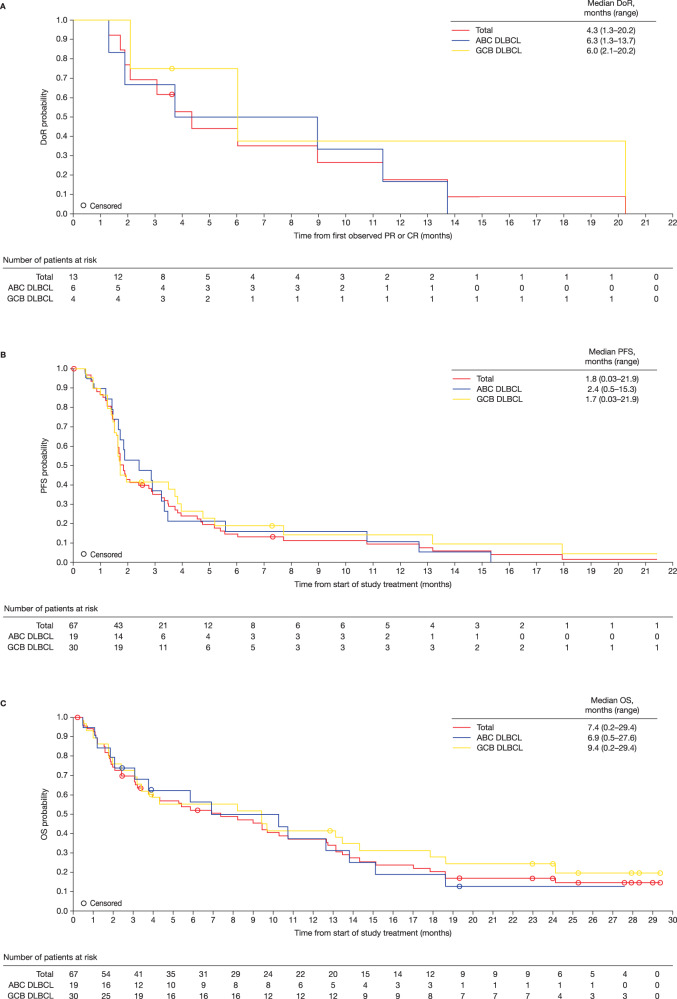
Kaplan–Meier curves. **a** Median overall DoR; **b** Median PFS; and **c** Median OS (overall cohort). ABC activated B-cell like, DLBCL diffuse large B-cell lymphoma, DoR duration of response, GCB germinal center B-cell like, PFS progression-free survival, OS overall survival.

Nineteen patients had ABC DLBCL, 30 patients had GCB DLBCL, three patients were unclassifiable, and 15 patients did not have a valid assessment and were deemed missing due to lack of adequate patient material. For those patients evaluable for DLBCL COO, patients with ABC DLBCL had a numerically lower incidence of DLBCL transformed from FL (15.8% vs. 30.0%), a higher incidence of stage IV disease (78.9% vs. 46.7%), a shorter median time from diagnosis to start of copanlisib treatment (16.8 months vs. 41.4 months), and had received numerically fewer prior lines of therapy (2 vs. 3.5) compared with patients with GCB DLBCL (Table 1). A similar proportion of patients with ABC DLBCL and GCB DLBCL were refractory against the last systemic therapy (78.9% vs. 80.0%) (Fig. 1).

NGS data were available for 54 patients, 45 of whom had wild-type *CD79B* and nine of whom had mutant *CD79B*; NGS data were missing for 13 patients due to lack of adequate material.

Forty patients comprised the PPS (Supplementary Table 1). The main reason for exclusion (18/27 patients) was lack of baseline COO assessment and/or *CD79B* mutational assessment due to lack of adequate material. Of the 40 patients in the PPS, 16 had ABC DLBCL, six of whom had mutant *CD79B*. In addition, 22 patients had GCB DLBCL, two of whom had mutant *CD79B*. Two patients had an unclassifiable DLBCL subtype. Ten patients had DLBCL transformed from FL.

Median overall extent of exposure was 1.6 months (range 0.2–16.6), corresponding to a median of 1.75 cycles (range 0.3–18.0). Patients received a median of six infusions (range 1–54). At the database cutoff, no patients were ongoing with study treatment.

**Fig. 2:**
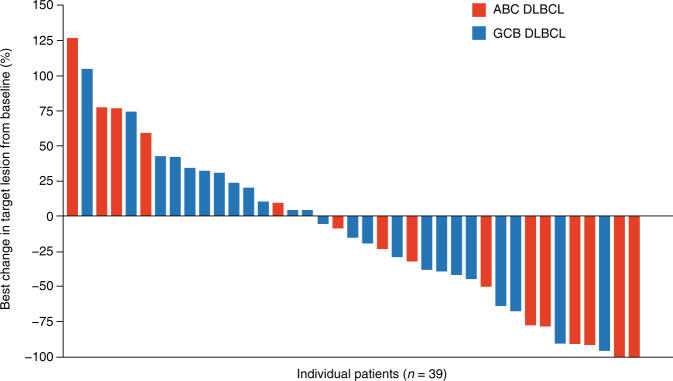
Waterfall plot of percentage best change in target lesion size from baseline (investigator assessment) in patients with ABC DLBCL and GCB DLBCL (overall cohort). ABC activated B-cell like, DLBCL diffuse large B-cell lymphoma, GCB germinal center B-cell like.

In the overall cohort, the ORR was 19.4%: five patients (7.5%) achieved CR and eight patients (11.9%) achieved PR. The DCR was 40.3% (Table 2a). The ORR was 31.6% (6/19 patients) for ABC DLBCL patients and 13.3% (4/30 patients) for GCB DLBCL patients (*P* = 0.1413) or, similarly, 15.2% (5/33 patients) for GCB DLBCL plus unclassifiable patients (*P* = 0.1751). Accordingly, GCB DLBCL patients had an 18.5% lower ORR than ABC DLBCL plus unclassifiable patients (*P* = 0.1329) (Table 2b). Median DoR was 4.3 months (range 1.3–20.2) (6.3 [range 1.3–13.7] and 6.0 months [range 2.1–20.2] for patients with ABC DLBCL and GCB DLBCL, respectively) (Fig. 1a). Median duration of stable disease was 3.5 months (range 1.5–17.9). Of the 39 patients with calculable percentage changes from baseline in target lesions, 46.7% (7/15) and 16.7% (4/24) of ABC DLBCL and GCB DLBCL patients, respectively, had a ≥50% reduction in lesion size (Fig. 2).

**Table 2:** Tumor response based on investigator assessment. **a** Tumor response in the overall cohort; **b** objective response rate in molecular DLBCL subtypes in the overall cohort and PPS.

(A) Tumor response in the overall cohort								
	Total *N* = 67	*CD79B* mutational status *n* = 67			DLBCL COO subgroup *n* = 67			
		Mutant *CD79B* *n* = 9	Wild-type *CD79B* *n* = 45	Missing *n* = 13	ABC DLBCL *n* = 19	GCB DLBCL *n* = 30	Unclassifiable *n* = 3	Missing *n* = 15
Best overall response, *n* (%)								
CR	5 (7.5)	1 (11.1)	4 (8.9)	0	4 (21.1)	1 (3.3)	0	0
PR	8 (11.9)	1 (11.1)	5 (11.1)	2 (15.4)	2 (10.5)	3 (10.0)	1 (33.3)	2 (13.3)
Stable disease	14 (20.9)	3 (33.3)	9 (20.0)	2 (15.4)	4 (21.1)	8 (26.7)	0	2 (13.3)
Progressive disease	30 (44.8)	4 (44.4)	17 (37.8)	9 (69.2)	7 (36.8)	13 (43.3)	2 (66.7)	8 (53.3)
Not evaluable/not available^a^	10 (14.9)	0	10 (22.2)	0	2 (10.5)	5 (16.7)	0	3 (20.0)
ORR, *n* (%)	13 (19.4)	2 (22.2)	9 (20.0)	2 (15.4)	6 (31.6)	4 (13.3)	1 (33.3)	2 (13.3)
90% CI	11.9, 29.1	4.1, 55.0	10.9, 32.3	2.8, 41.0	14.7, 53.0	4.7, 28.0	1.7, 86.5	2.4, 36.3
DCR, *n* (%)	27 (40.3)	5 (55.6)	18 (40.0)	4 (30.8)	10 (52.6)	12 (40.0)	1 (33.3)	4 (26.7)

(B) Objective response rate in molecular DLBCL subtypes in the overall cohort and PPS						
Biomarker	Overall cohort			PPS		
	ORR, %	ORR difference, % (90% CI)	*P* value	ORR, %	ORR difference, % (90% CI)	*P* value
ABC vs. GCB	31.6 vs. 13.3	18.2 (−6.1, 41.0)	0.1413	37.5 vs. 13.6	23.9 (−3.3, 48.3)	0.1074
ABC vs. GCB plus unclassifiable	31.6 vs. 15.2	16.4 (−7.2, 39.1)	0.1751	37.5 vs. 16.7	20.8 (−6.8, 46.2)	0.1638
GCB vs. ABC plus unclassifiable	13.3 vs. 31.8	−18.5 (−40.1, 4.6)	0.1329	13.6 vs. 38.9	−25.3 (−49.1, 1.1)	0.0762
Unclassifiable^b^	33.3			50.0		

*ABC* activated B-cell like, *CI* confidence interval, *COO* cell of origin, *CR* complete response, *DLBCL* diffuse large B-cell lymphoma, *GCB* germinal center B-cell like, *ORR* objective response rate, *PPS* per-protocol set, *PR* partial response.

^a^One patient had postbaseline tumor assessment(s) that could not be evaluated by the investigator and nine patients had no postbaseline tumor assessment due to discontinuation because of progression by clinical judgement, death occurring before disease was reevaluated, or other reasons; these ten patients were considered nonresponders.

^b^Overall cohort, *n* = 3; PPS, *n* = 2; no statistical comparisons reported due to small sample size.

For patients with mutant *CD79B*, the ORR was 22.2% (2/9 patients); for those with wild-type *CD79B*, ORR was 20.0% (9/45 patients) (Table 2a). Of the patients who had DLBCL transformed from FL, ORR was 21.4% (3/14 patients: one CR; two PR).

Analysis of ORR in the PPS subgroup of patients with more complete molecular profiling information available demonstrated similar results to the overall cohort (Table 2b, Supplementary Table 2). Comparing pairwise differences for ORR in the PPS subgroups, ABC DLBCL patients had a response rate of 37.5%, whereas GCB DLBCL patients had a response rate of 13.6%. ABC DLBCL patients had a 20.8% higher ORR than GCB DLBCL plus unclassifiable patients (*P* = 0.1638), and GCB DLBCL patients had an ORR 25.3% lower than ABC DLBCL plus unclassifiable patients (*P* = 0.0762). Median DoR in the PPS subgroup was 3.7 months (range 1.3–20.2) (6.3 [range 1.3–13.7] and 20.2 months [range 2.1–20.2] for patients with ABC DLBCL and GCB DLBCL, respectively) (Supplementary Fig. 1a).

The median overall PFS in the overall cohort was 1.8 months (range 0.03–21.9). Estimated PFS rates at 3, 6, 9, and 12 months were 34.7%, 14.2%, 10.8%, and 9.0%, respectively. Median PFS was 2.4 months (range 0.5–15.3) for ABC DLBCL patients and 1.7 months (range 0.03–21.9) for GCB DLBCL patients (Fig. 1b). Median OS was 7.4 months (range 0.2–29.4) (Fig. 1c), and estimated OS rates at 3, 6, 9, and 12 months were 69.7%, 52.2%, 45.4%, and 37.0%, respectively.

Median overall PFS in the PPS subgroup was 2.8 months (range 0.7–21.9), with estimated PFS rates of 44.6%, 18.4%, and 15.3% at 3, 6, and 12 months, respectively (Supplementary Fig. 1b). Median PFS was 2.9 months (range 0.7–15.3) for ABC DLBCL patients and 1.8 months (range 1.2–21.9) for GCB DLBCL patients.

**Fig. 3:**
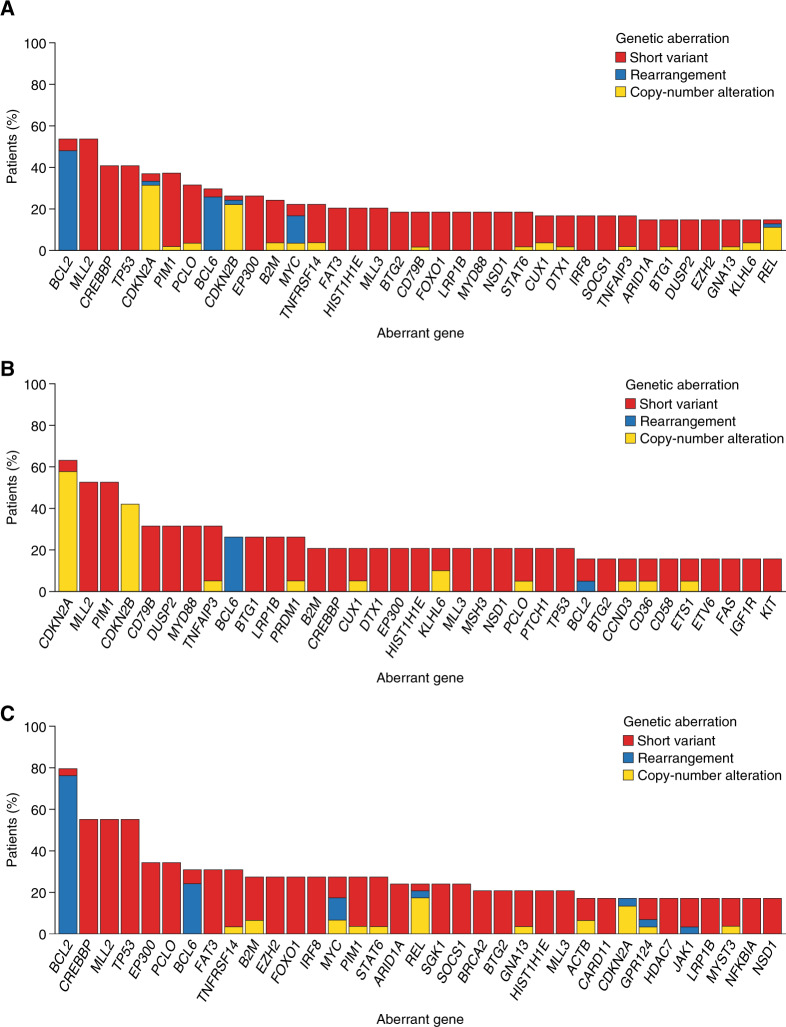
Most common tumor aberrations detected. **a** The overall cohort; **b** ABC DLBCL (overall cohort); and **c** GCB DLBCL (overall cohort). ABC activated B-cell like, DLBCL diffuse large B-cell lymphoma, GCB germinal center B-cell like.

Of the 54 patient samples with targeted NGS data available, 338 genes with aberrations were detected. Samples had a median of 24.5 (range 5–61) alterations detected by NGS. Overall, the most common alterations included *BCL2* (29 patient samples, 53.7%; predominantly gene rearrangements), *MLL2* (29 patient samples, 53.7%), *TP53* (22 patient samples, 40.7%), *BCL6* (16 patient samples, 29.6%; mainly gene rearrangements with a few short variants), and *MYC* (12 patient samples, 22.2%; mostly rearrangements, some short variants and copy-number alterations) (Fig. 3a), with differences observed between the COO groups (Fig. 3b, c, Supplementary Table 3, Supplementary Fig. 2).

**Fig. 4:**
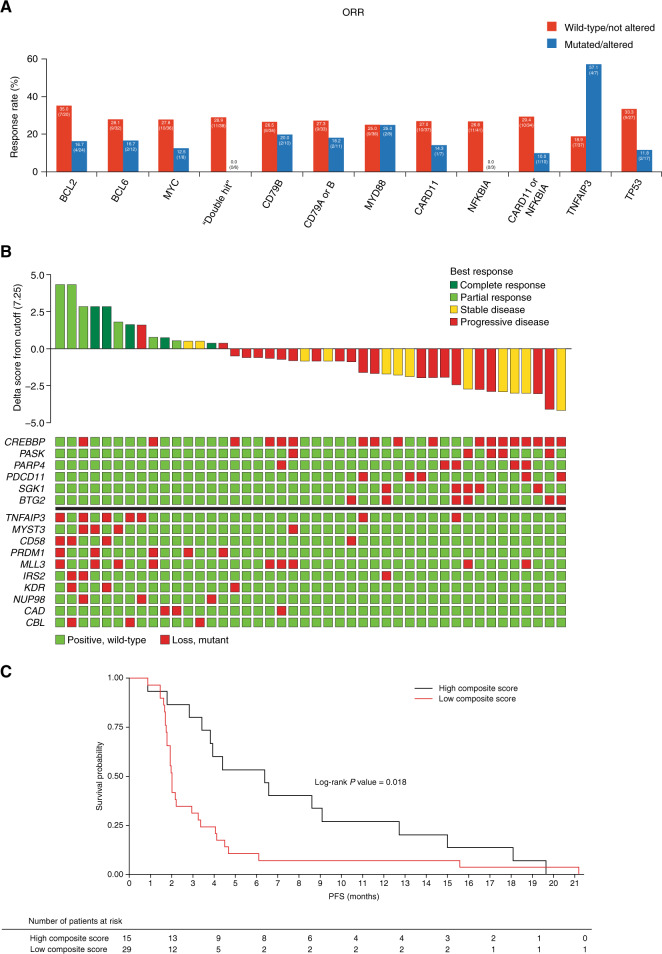
Correlation of NGS biomarker data with response. **a** Correlation of mutational status and clinical outcome (all patients with NGS and response data were analyzed; *n * =  44). **b** Multigene mutation signature separating responders from nonresponders and their computed composite scores (patients are sorted based on the composite score. Score values are shown as a waterfall of delta scores to the cutoff level. The list of genes with mutations predictive of response is given in the matrix below the waterfall plot. Genes with the presence of a mutation in the responder group [and absence of mutation in the nonresponder groups] contributing to the score are shown on the bottom, while genes with the absence of a mutation in the responder group [or presence of mutation in the nonresponder group] contributing to the score are shown on top) and **c** PFS by mutation composite score. NGS next-generation sequencing, ORR objective response rate, PFS progression-free survival.

Forty-four patients had both NGS biomarker data and investigator-assessed response data available (Fig. 4a, Supplementary Table 4). Of genes known to be associated with constitutive oncogenic nuclear factor-κB signaling, the ORR for patients with and without *MYD88* mutations was 25.0% (2/8 patients and 9/36 patients, respectively), while patients with mutated *TNFAIP3* demonstrated a better ORR than those with wild-type (57.1% [4/7 patients] vs. 18.9% [7/37 patients]; *P* = 0.054). Patients with *NFKBIA* mutations had worse outcomes than those with wild-type *NFKBIA* (PFS: hazard ratio 0.21, *P* = 0.013; DCR: 0% [0/3 patients] vs. 56.1% [23/41 patients], *P* = 0.100). *CARD11* wild-type and *CARD11-*mutant patients had ORRs of 27.0% (10/37 patients) and 14.3% (1/7 patients), respectively, and DCRs of 56.8% (21/37 patients) and 28.6% (2/7 patients), respectively.

Response rates for patients with and without *BCL2* were 16.7% (4/24 patients) and 35.0% (7/20 patients), respectively. Of the six patients with alterations in both *MYC* and either *BCL2* or *BCL6*, no responders were identified (Fig. 4a, Supplementary Table 4), compared with an ORR of 28.9% (11/38 patients) in patients without both alterations. While the group sizes in most of these exploratory analyses are small, the findings suggest hypotheses that can be investigated in future studies.

To better understand these observed responses to copanlisib, we performed an unbiased exploratory analysis that identified a 16-gene mutation signature separating responders from nonresponders (Fig. 4b). A composite score was calculated, reflecting numerically the collective presence of mutations in specific genes in our resulting gene set, along with the absence of mutations in others. Patients with a high composite score had a higher ORR and longer PFS compared with those with a low composite score (ORR 11/15 patients with a composite score above the cutoff who had either PR or CR, compared with 0/29 patients with a score below the cutoff who had a response; PFS 5.4 vs. 1.7 months; hazard ratio 2.27 [95% CI 1.15, 4.49]; *P* = 0.018) (Fig. 4c). The genes in this signature included several with higher prevalence in one molecular subtype (ABC DLBCL vs. GCB DLBCL) than the other (e.g., *TNFAIP3*, *CD58*, *PRDM1*, and *CBL* in ABC DLBCL and *MYST3*, *CREBBP*, *SGK1*, *PARP4*, and *PDCD11* in GCB DLBCL) (Fig. 4b, Supplementary Fig. 2), as well as several genes with known prognostic significance in DLBCL (*TNFAIP3*, *CD58*, *CREBBP*, and *PRDM1*).

Overall, the safety analysis set comprised 67 patients; 65 patients (97.0%) experienced at least one treatment-emergent AE (TEAE) (Table 3). The most common grade 3 TEAEs were hypertension (32.8%) and hyperglycemia (22.3%), and the most common grade 4 event was hyperglycemia (9.0%). Most postinfusion hypertension events were grade ≤2 and were transient, manageable, and resolved following treatment; no grade 4 hypertension events were reported. Results were similar in patients with and without a medical history of hypertension. The prevalence of postinfusion hyperglycemia events was highest in cycle 1, after which new hyperglycemia events occurred at a lower rate (80.6%, 46.3%, and 16.4% on day 1 of cycles 1, 2, and 3, respectively; data not shown for remaining cycles).

**Table 3:** Overview of TEAEs and incidence of TEAEs and drug-related TEAEs occurring in ≥10% of the total population (overall cohort).

*n* (%)	Grade 1 or 2	Grade 3 or 4	Grade 5	Total *N* = 67
Any TEAE^a^	7 (10.4)	44 (65.7)	14 (20.9)	65 (97.0)
SAEs	2 (3.0)	28 (41.8)	14 (20.9)	44 (65.7)
Patients with TEAEs leading to dose reduction	2 (3.0)	7 (10.4)	0	9 (13.4)
Patients with TEAEs leading to dose interruption/delay	11 (16.4)	23 (34.3)	0	34 (50.7)
Patients with TEAEs leading to permanent discontinuation	4 (6.0)	13 (19.4)	0	17 (25.4)
Incidence of TEAEs occurring in >10% of the total population				
Hypertension	5 (7.5)	22 (32.8)	0	27 (40.3)
Diarrhea	24 (35.8)	1 (1.5)	0	25 (37.3)
Hyperglycemia	1 (1.5)	21 (31.3)	0	22 (32.8)
Nausea	20 (29.9)	1 (1.5)	0	21 (31.3)
Fatigue	18 (26.9)	0	1 (1.5)^b^	19 (28.4)
Pyrexia	13 (19.4)	1 (1.5)	0	14 (20.9)
Cough	11 (16.4)	1 (1.5)	0	12 (17.9)
Vomiting	11 (16.4)	1 (1.5)	0	12 (17.9)
Constipation	11 (16.4)	0	0	11 (16.4)
Decreased appetite	10 (14.9)	0	0	10 (14.9)
Deterioration in general physical health	0	1 (1.5)	8 (11.9)	9 (13.4)
Headache	9 (13.4)	0	0	9 (13.4)
Neutropenia	1 (1.5)	8 (11.9)	0	9 (13.4)
Rash	8 (11.9)	1 (1.5)	0	9 (13.4)
Hypokalemia	4 (6.0)	4 (6.0)	0	8 (11.9)
Mouth ulceration	7 (10.4)	1 (1.5)	0	8 (11.9)
Dyspnea	4 (6.0)	3 (4.5)	0	7 (10.4)
Peripheral edema	7 (10.4)	0	0	7 (10.4)
Incidence of drug-related TEAEs occurring in >10% of the total population				
Hypertension	4 (6.0)	19 (28.4)	0	23 (34.3)
Hyperglycemia	1 (1.5)	20 (29.9)	0	21 (31.3)
Fatigue	12 (17.9)	0	0	12 (17.9)
Nausea	12 (17.9)	0	0	12 (17.9)
Diarrhea	10 (14.9)	1 (1.5)	0	11 (16.4)
Vomiting	7 (10.4)	1 (1.5)	0	8 (11.9)
Mouth ulceration	6 (9.0)	1 (1.5)	0	7 (10.4)
Neutropenia	1 (1.5)	6 (9.0)	0	7 (10.4)

*SAE* serious adverse event, *TEAE* treatment-emergent adverse event.

^a^Specified event starting or worsening between start of treatment and 30 days after the end of treatment.

^b^This patient received treatment with copanlisib for 14 days and was withdrawn from the study due to disease progression. The patient experienced increasing fatigue that was considered an SAE due to hospitalization occurring 15 days after the last dose of copanlisib, with a reported grade 5 (fatal) outcome occurring 2 days later, considered as a symptom of disease progression. The event was considered unrelated to treatment with copanlisib or protocol-required procedures, with the primary cause of death reported as underlying disease.

Serious AEs (SAEs) occurred in 44 patients (65.7%). Twenty-two patients (32.8%) experienced grade 3 SAEs, six patients (9.0%) experienced grade 4 SAEs, and all 14 grade 5 events (20.9%) were considered SAEs. Fifty-five patients (82.1%) experienced drug-related TEAEs, most commonly hypertension (34.3%), hyperglycemia (31.3%), and fatigue and nausea (17.9% each) (Table 3). Three patients (4.5%) experienced pneumonitis (two patients [3.0%] with grade 3 and one patient [1.5%] with grade 4), all considered drug related. Five patients (7.5%) experienced a drug-related infection or infestation of grade ≥3. One patient experienced drug-related grade 2 *Clostridium colitis*. The most common laboratory abnormalities were hyperglycemia (63/67, 94.0%), anemia (61/65, 93.8%), lymphocytopenia (54/65, 83.1%), thrombocytopenia (38/65, 58.5%), leukocytopenia (37/65, 56.9%), and neutropenia (29/65, 44.6%).

Hyperglycemia TEAEs were present at a higher incidence in the 11 patients with a confirmed diagnosis of diabetes compared with patients without a history of diabetes, with grade 3 hyperglycemia experienced by 63.6% (7/11) and 21.4% (12/56) of patients, respectively. Grade 4 hyperglycemia was experienced by 27.3% (3/11) and 5.4% (3/56) of patients, respectively. Mean change in glycated hemoglobin from baseline was slightly higher in diabetic patients (1.50 ± 0.85%; *n* = 8) than in nondiabetic patients (0.49 ± 0.98%; *n* = 41). Most postinfusion hyperglycemia events after cycle 1, day 1 were grade <2, with no grade 4 events reported in any patients.

TEAEs leading to permanent discontinuation occurred in 25.4% of patients and were considered drug related in 11.9% (Table 3), including one patient (1.5%) each with asthenia, thrombocytopenia, hyperglycemia, hyponatremia, hypophosphatemia, pneumonitis, rash, and Stevens–Johnson syndrome. No patient discontinued treatment due to hypertension. One patient each discontinued therapy due to hyperglycemia and pneumonitis. Fourteen grade 5 TEAEs (20.9%) were reported. None of these were considered drug related. Patient deaths were mostly due to disease progression (43 patients, 64.2%).

Dose interruptions or delays, 91% of which were due to AEs, were experienced by 38 patients (56.7%). Of these dose interruptions or delays, 94% lasted ≤1 week (median 0.79 weeks [range 0–2.0]) and nine patients (13.4%) had a dose reduction to 45 mg, mostly due to AEs. The most common reason for treatment discontinuation was radiologic or clinical progressive disease (49/67, 73.1%).

## Discussion

For patients with relapsed/refractory DLBCL, treatment options are still very limited, presenting an urgent need for novel therapeutic approaches. In this phase II study, monotherapy treatment with copanlisib demonstrated an ORR of 19.4%, a response comparable with response rates reported with other nonchemotherapy single agents in unselected DLBCL, including the immunomodulatory agent lenalidomide (27.5%) [25] and the Bruton’s tyrosine kinase inhibitor ibrutinib (25%) [26].

These clinical data reflect previous preclinical in vitro and in vivo studies that have suggested stronger activity of PI3K-α/-δ inhibition in ABC DLBCL compared with GCB DLBCL models [13–15, 17]. The results are generally consistent with these findings, with copanlisib demonstrating a numerically higher response rate in ABC DLBCL compared with GCB DLBCL patients. In the overall cohort, patients with ABC DLBCL demonstrated an ORR of 31.6%, compared with 13.3% in patients with GCB DLBCL, though the difference was not statistically significant (*P* = 0.1413). These data suggest that despite the worse prognosis expected for this group, ABC DLBCL may respond preferentially to pharmacologic inhibition of oncogenic PI3K signaling. The results of our study are comparable to those reported for ibrutinib (response rate of 37% for ABC DLBCL and 5% for GCB DLBCL) and lenalidomide (response rate of 46% for ABC DLBCL and 21.4% for GCB DLBCL) [25, 26].

Copanlisib elicited similar responses in patients with and without *CD79B* mutations, indicating modest antilymphoma activity regardless of the presence of chronic BCR signaling. These results echo those from a trial investigating the efficacy of ibrutinib monotherapy in relapsed/refractory DLBCL patients, in which patients with mutated and wild-type *CD79B* responded similarly to treatment [26]. Further exploratory mutational analyses highlighted that several mutations modifying oncogenic nuclear factor-κB pathway signaling may have an impact on the efficacy of copanlisib. While we determined that *MYD88* mutations do not influence response, the mutational status of *TNFAIP3*, *NFKBIA*, and *CARD11* might be associated with sensitivity to copanlisib. In addition, genetic aberrations in other genes seem to influence response to copanlisib. Patients with *BCL2* mutations had a numerically lower response rate, and no patient with alterations in *MYC* plus either *BCL2* or *BCL6* achieved an objective response.

Using an unbiased approach, we were able to identify a 16-gene mutation signature separating copanlisib responders from nonresponders. Though exploratory in nature, this signature might be utilized in the future to select patients who are treated with copanlisib. Though not all genes in the signature have a known prognostic role in DLBCL, it cannot be ruled out that the signature may be separating prognostic groups, where patients with good prognosis may have a higher likelihood of responding to therapy than those with poor prognosis, which could result in a better response rate and longer PFS. However, whether these findings reflect prognostic influence conferred by these mutations or a predictive ability for copanlisib activity could not be determined in the present study due to the small groups and lack of a control arm. These results provide the basis for future validation and analyses.

Recently, several large genetic studies identified several novel molecular DLBCL subtypes [27–29]. One of these studies identified five DLBCL subsets based on recurrent mutations, somatic copy-number alterations, structural variants, and defined coordinate signatures [27]. Another study described four prominent genetic subtypes in DLBCL, termed MCD (based on the co-occurrence of *MYD88L265P* and *CD79B* mutations), BN2 (based on *BCL6* fusions and *NOTCH2* mutations), N1 (based on *NOTCH1* mutations), and EZB (based on *EZH2* mutations and *BCL2* translocations), using exome and transcriptome sequencing, array-based DNA copy-number analysis, and targeted amplicon resequencing [29]. It will be interesting to investigate in the future if these specific molecular subsets of DLBCL will respond preferentially to copanlisib or other PI3K inhibitors.

Overall, copanlisib demonstrated a safety profile that was recognizable and manageable. TEAEs most commonly reported were hypertension, diarrhea, and hyperglycemia, consistent with toxicities observed with copanlisib in previous trials [19, 20, 30]. Hyperglycemia events were generally grade <4. Hyperglycemia is likely to be an on-target effect of the inhibition of the PI3K-α isoform, related to downstream signaling of insulin receptors, leading to transient, reduced tissue utilization of glucose, and/or insulin resistance [31]. Hypertension events were never grade 4 in severity and did not lead to discontinuation in any of the affected patients. The molecular mechanisms of copanlisib leading to hypertension are currently unclear. However, the majority of hypertension events observed in patients treated with copanlisib were transient, which may be related to acute vasoconstriction resulting from the intravenous infusion of copanlisib [32]. Overall, hyperglycemia and hypertension were transient and considered manageable, with almost all patients reporting values approaching baseline prior to subsequent infusions, consistent with previous pharmacodynamic studies [30].

In conclusion, single-agent copanlisib demonstrated modest antilymphoma activity in patients with relapsed/refractory DLBCL with a numerically higher response rate in patients with ABC DLBCL vs. patients with GCB DLBCL, with similar activity in patients with and without *CD79B* mutations. A 16-gene mutation signature associated with improved outcomes was identified as a possible approach (with further validation) for selecting DLBCL patients for treatment with copanlisib. Copanlisib showed an acceptable safety profile, with the most common toxicities of hypertension and hyperglycemia being transient and manageable. To our knowledge, this is the largest phase II study on the use of a PI3K inhibitor in patients with DLBCL; therefore, further exploration of copanlisib in combination with other targeted therapies (e.g., a Bruton’s tyrosine kinase inhibitor [14] or a BCL2 antagonist [15]) or in molecularly defined subgroups of DLBCL is warranted. In addition, a phase II study evaluating copanlisib in combination with rituximab-CHOP chemotherapy in previously untreated patients with DLBCL will be initiated in 2020 (EudraCT 2018-003560-31).

### Supplementary information

Supplemental Material

## Data Availability

Availability of the data underlying this publication will be determined according to Bayer’s commitment to the EFPIA/PhRMA “Principles for responsible clinical trial data sharing”. This pertains to scope, time point, and process of data access. As such, Bayer commits to sharing upon request from qualified scientific and medical researchers patient-level clinical trial data, study-level clinical trial data, and protocols from clinical trials in patients for medicines and indications approved in the United States (US) and European Union (EU) as necessary for conducting legitimate research. This applies to data on new medicines and indications that have been approved by the EU and US regulatory agencies on or after January 1, 2014. Interested researchers can use www.clinicalstudydatarequest.com to request access to anonymized patient-level data and supporting documents from clinical studies to conduct further research that can help advance medical science or improve patient care. Information on the Bayer criteria for listing studies and other relevant information is provided in the study sponsors section of the portal. Data access will be granted to anonymized patient-level data, protocols, and clinical study reports after approval by an independent scientific review panel. Bayer is not involved in the decisions made by the independent review panel. Bayer will take all necessary measures to ensure that patient privacy is safeguarded.
