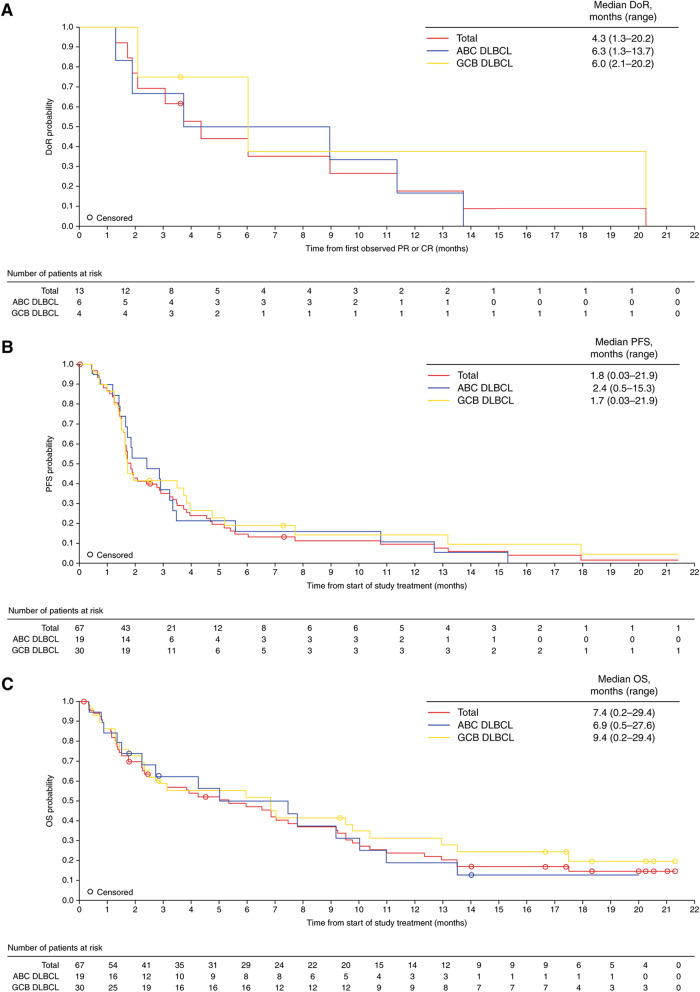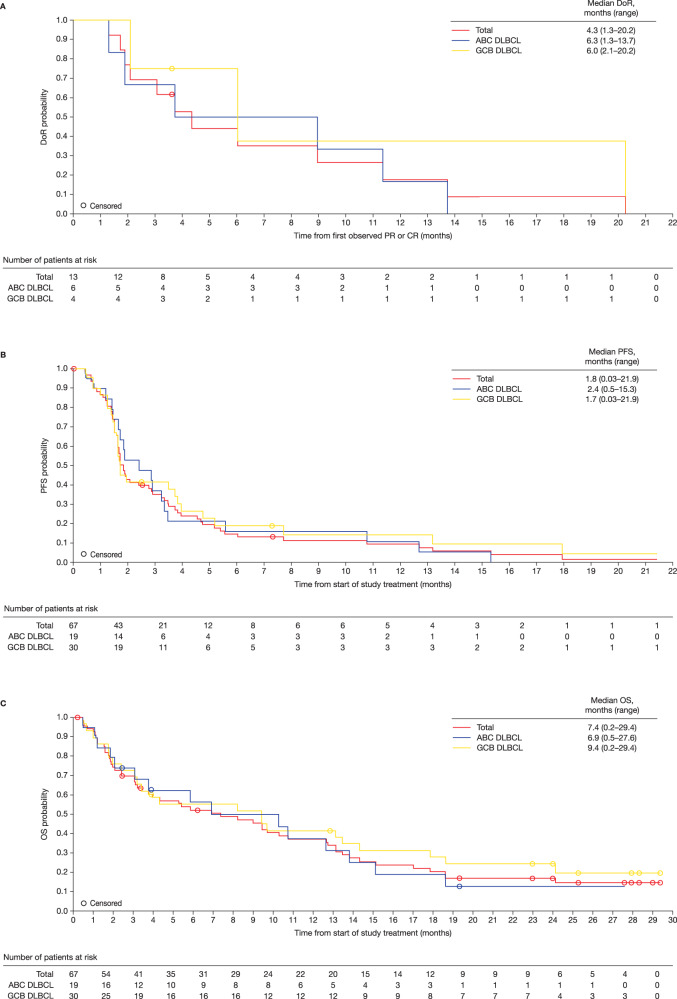# Correction: Single-agent activity of phosphatidylinositol 3-kinase inhibition with copanlisib in patients with molecularly defined relapsed or refractory diffuse large B-cell lymphoma

**DOI:** 10.1038/s41375-023-02133-2

**Published:** 2024-01-10

**Authors:** Georg Lenz, Eliza Hawkes, Gregor Verhoef, Corinne Haioun, Soon Thye Lim, Dae Seog Heo, Kirit Ardeshna, Geoffrey Chong, Jacob Haaber, Wei Shi, Igor Gorbatchevsky, Susanne Lippert, Florian Hiemeyer, Paolo Piraino, Georg Beckmann, Carol Peña, Viktoriya Buvaylo, Barrett H. Childs, Gilles Salles

**Affiliations:** 1 Department of Medicine A, Hematology, Oncology, and Pneumology, University Hospital Münster, Münster, Germany; 2 Eastern Health Clinical School, Monash University, Olivia Newton John Cancer Research and Wellness Centre, Melbourne, VIC Australia; 3 University Hospitals Leuven, Leuven, Belgium; 4 Lymphoid Malignancies Unit, Groupe Hospitalier Henri Mondor-Albert Chenevier, Creteil, France; 5 National Cancer Centre Singapore and Duke-NUS Medical School, Singapore, Singapore; 6 Department of Internal Medicine, Seoul National University Hospital, Seoul, South Korea; 7 University College London Hospitals NHS Foundation Trust, London, UK; 8 Ballarat Regional Integrated Cancer Centre, Ballarat, VIC Australia; 9 Department of Hematology, Odense University Hospital, Odense, Denmark; 10 Bayer China, Beijing, China; 11 Bayer HealthCare Pharmaceuticals, Inc., Whippany, NJ USA; 12 Pharmaceuticals Division, Bayer AG, Berlin, Germany; 13 Hospices Civils de Lyon, Université de Lyon, Centre Hospitalier Lyon-Sud, Service d’hématologie, Lyon, France

**Keywords:** B-cell lymphoma, Targeted therapies

Correction to: *Leukemia* 10.1038/s41375-020-0743-y, published online 14 February 2020

In this article, Figure 1 was incorrect.

The updated figure can be found below.

The original article has been corrected.