# Supplementary material for: Single-agent activity of phosphatidylinositol 3-kinase inhibition with copanlisib in patients with molecularly defined relapsed or refractory diffuse large B-cell lymphoma
Source: Leukemia. 2020 Feb 14;34(8):2184–97. doi: 10.1038/s41375-020-0743-y (PMC7387311; doi:10.1038/s41375-020-0743-y)
Supplement: Supplementary file 1 — Supplemental Material [file 41375_2020_743_MOESM1_ESM.docx]

**Supplementary information**

**Methods**

***Additional inclusion and exclusion criteria***

Additional eligibility criteria included the availability of a fresh and/or archival tumor biopsy collected after the last relapse of disease, measurable disease, an Eastern Cooperative Oncology Group performance status of ≤2, and adequate bone marrow, liver, and renal function. Key exclusion criteria included active Common Terminology Criteria for Adverse Events grade 3/4 infection, previous therapy with copanlisib or other phosphatidylinositol 3-kinase inhibitors, interstitial lung disease of any severity and/or severely impaired lung function, type I and II diabetes mellitus with glycated hemoglobin >8.5%, uncontrolled arterial hypertension, and New York Heart Association class III or IV heart disease.

***Definitions of secondary efficacy endpoints***

Duration of response was defined as the time from the first observed objective response until radiologic disease progression or death. Progression-free survival was defined as the time from first copanlisib administration until radiologic disease progression or death. Overall survival was defined as the time from first copanlisib administration until death. Duration of stable disease was evaluated in patients failing to achieve a best response of complete response or partial response, but who achieved stable disease, and was defined as the time from the start of copanlisib treatment until radiologic disease progression or death. Disease control rate was defined as the proportion of patients who had a best response of complete response, partial response, or stable disease.

***Statistical sample size planning***

The primary evaluation was to compare objective response rate between *CD79B* status and between diffuse large B-cell lymphoma cell of origin status. The statistical sample size planning of this exploratory trial aimed to include at least 10 evaluable patients with *CD79B* mutation, requiring enrollment of 66 patients assuming a *CD79B* mutation rate of 16.7% and a nonevaluable rate of 10%.

The response rate in patients with *CD79B* mutations was assumed to be approximately 45%, and approximately 5% in wild-type patients, resulting in an assumed difference of 40 percentage points. For a difference of 40 percentage points, the exact two-sided 90% confidence interval for the difference most probably observed in the 10 evaluable patients with *CD79B* mutations ranged from 17–65%, with a length of approximately 50 percentage points, based on Santner and Snell methodology (1). This was considered sufficient to assess whether *CD79B* mutation status and/or cell of origin was a potential predictive biomarker for efficacy of copanlisib in diffuse large B-cell lymphoma. No accounting of multiplicity was performed.

***Additional details of the exploratory analysis of mutation-based signature***

Since there was no control arm in this study, a correspondence discriminant analysis was used to indicate the set of genes possibly associated with a specific outcome in the presence of copanlisib. This approach is a multivariate dimensionality reduction technique to reduce wide tables into fewer dimensions. To account for the small sample size and imbalance between classes, correspondence discriminant analysis was performed 1000 times on a noise-added data set obtained by resampling with replacement (bootstrapping) the mutation for each gene in the responder and nonresponder groups, obtaining a set of 50 pseudo-patients for responders and 50 for nonresponders. An arbitrary cutoff of 0.25 on the mean discriminant power obtained from the first factors of the 1000 repetitions was used to identify genes with frequencies most associated with the separation of responders and nonresponders.

A composite score was calculated, numerically reflecting the collective presence of mutations in certain genes in the gene set, along with the absence of mutations in others, with the mean discrimination power of the first factor used as weight in the formula:

$${Score}_{j}=\sum_{i=1}^{n} {WeightedPresence}_{i}+ \sum_{i=1}^{n} {WeightedAbsence}_{i}$$

*i* selected genes; *j* patient index

A cutoff of 7.25, chosen based on visual inspection, was used to separate those with a high composite score from those with a low composite score.

**Reference**

1. Santner TJ, Snell MK. Small-sample confidence intervals for *p*_1_−*p*_2_ and *p*_1_/*p*_2_ in 2 × 2 contingency tables. J Am Stat Assoc. 1980;75:386–94.

**Supplementary Table 1** Patient demographics and baseline characteristics in ABC DLBCL and GCB DLBCL in the PPS cohort

|  | **ABC DLBCL *n* = 16** | **GCB DLBCL *n* = 22** | **Total *N* = 40^a^** |
| --- | --- | --- | --- |
| Age (years), median (range) | 73.5 (57–84) | 67.5 (32–85) | 71.5 (32–85) |
| Age ≥65 years, *n* (%) | 14 (87.5) | 14 (63.6) | 30 (75.0) |
| Male, *n* (%) | 9 (56.3) | 15 (68.2) | 25 (62.5) |
| ECOG performance status, *n* (%) |  |  |  |
| 0 | 5 (31.3) | 7 (31.8) | 12 (30.0) |
| 1 | 8 (50.0) | 12 (54.5) | 22 (55.0) |
| 2 | 3 (18.8) | 3 (13.6) | 6 (15.0) |
| Histology of tumor, *n* (%) |  |  |  |
| DLBCL transformed from FL | 3 (18.8) | 7 (31.8) | 10 (25.0) |
| DLBCL not otherwise specified | 13 (81.3) | 15 (68.2) | 30 (75.0) |
| EBV-positive DLBCL of the elderly | 0 | 0 | 0 |
| T-cell/histocyte-rich large B-cell lymphoma | 0 | 0 | 0 |
| Stage at study entry, *n* (%) |  |  |  |
| I | 0 | 3 (13.6) | 3 (7.5) |
| II | 0 | 5 (22.7) | 5 (12.5) |
| III | 4 (25.0) | 6 (27.3) | 10 (25.0) |
| IV | 12 (75.0) | 8 (36.4) | 22 (55.0) |
| Median time from initial diagnosis to start of study treatment, months (range) | 19.5 (1.4–123) | 42.2 (0.7–192) | 38.2 (0.7–192) |
| Median time since first progression, months (range) | 3.1 (0.5–60.5) | 27.8 (0.7–184) | 5.2 (0.5–184) |
| Median time from most recent progression to start of study treatment,  months (range) | 1.0 (0.2–3.0) | 1.4 (0.2–6.2) | 1.2 (0.2–6.2) |
| Median prior anticancer therapy lines, *n* (range) | 2 (1–5) | 4 (1–13) | 3 (1–13) |
| Median time since last systemic anticancer therapy, months (range) | 1.9 (0.7–118) | 1.9 (1.0–68.5) | 2.0 (0.69–118) |
| Refractory against last systemic anticancer therapy, *n* (%) |  |  |  |
| Yes | 12 (75.0) | 16 (72.7) | 28 (70.0) |
| No | 4 (25.0) | 6 (27.3) | 12 (30.0) |

^a^Includes two unclassifiable patients

*ABC* activated B-cell like; *COO* cell of origin; *DLBCL* diffuse large B-cell lymphoma; *EBV* Epstein−Barr virus; *ECOG* Eastern Cooperative Oncology Group; *FL* follicular lymphoma; *GCB* germinal center B-cell like; *PPS* per-protocol set

**Supplementary Table 2** Tumor response based on investigator assessment (PPS)

|  |  | ***CD79B* mutational status *n* = 40** | | **DLBCL COO subgroup *n* = 40** | | |
| --- | --- | --- | --- | --- | --- | --- |
|  | **Overall PPS *n* = 40** | **Mutant *CD79B* *n* = 8** | **Wild-type *CD79B* *n* = 32** | **ABC DLBCL *n* = 16** | **GCB DLBCL *n* = 22** | **Unclassifiable *n* = 2** |
| Best overall response, *n* (%) |  |  |  |  |  |  |
| Complete response | 5 (12.5) | 1 (12.5) | 4 (12.5) | 4 (25.0) | 1 (4.5) | 0 |
| Partial response | 5 (12.5) | 1 (12.5) | 4 (12.5) | 2 (12.5) | 2 (9.1) | 1 (50.0) |
| Stable disease | 12 (30.0) | 3 (37.5) | 9 (28.1) | 4 (25.0) | 8 (36.4) | 0 |
| Progressive disease | 16 (40.0) | 3 (37.5) | 13 (40.6) | 5 (31.3) | 10 (45.5) | 1 (50.0) |
| Not evaluable/ not available | 2 (5.0) | 0 | 2 (6.3) | 1 (6.3) | 1 (4.5) | 0 |
| ORR, *n* (%)  90% CI | 10 (25.0)  14.2, 38.7 | 2 (25.0)  4.6, 60.0 | 8 (25.0)  13.1, 40.6 | 6 (37.5)  17.8, 60.9 | 3 (13.6)  3.8, 31.6 | 1 (50.0)  2.5, 97.5 |
| DCR, *n* (%) | 22 (55.0) | 5 (62.5) | 17 (53.1) | 10 (62.5) | 11 (50.0) | 1 (50.0) |

*ABC* activated B-cell like; *CI* confidence interval; *COO* cell of origin; *DCR* disease control rate; *DLBCL* diffuse large B-cell lymphoma; *GCB*  germinal center B-cell like; *ORR* objective response rate; *PPS* per-protocol set

**Supplementary Table 3** Gene mutation frequencies in ABC and GCB DLBCL patient samples (overall cohort)

| % (*n*) | Mutant/wild-type | ABC DLBCL *n* = 19 | GCB DLBCL *n* = 29 | Unclassifiable *n* = 2 | *P* value |
| --- | --- | --- | --- | --- | --- |
| *BCL2*^a^ | Mutant | 16 (3) | 79 (23) | 50 (1) | <0.001 |
|  | Wild-type | 84 (16) | 21 (6) | 50 (1) |  |
| *BCL6*^a^ | Mutant | 26 (5) | 31 (9) | 0 | 1 |
|  | Wild-type | 74 (14) | 69 (20) | 100 (2) |  |
| *MYC*^b^ | Mutant | 11 (2) | 28 (8) | 50 (1) | 0.276 |
|  | Wild-type | 89 (17) | 72 (21) | 50 (1) |  |
| *MYC* and either *BCL2* or *BCL6*^a^ | Mutant | 0 | 28 (8) | 50 (1) | 0.015 |
|  | Wild-type | 100 (19) | 72 (21) | 50 (1) |  |
| *MYC*, *BCL2*, and *BCL6*^a^ | Mutant | 0 | 3 (1) | 0 | 0.267 |
|  | Wild-type | 100 (19) | 97 (28) | 100 (2) |  |
| *CD79A* | Mutant | 5 (1) | 7 (2) | 0 | 1 |
|  | Wild-type | 95 (18) | 93 (27) | 100 (2) |  |
| *CD79B*^c^ | Mutant | 32 (6) | 10 (3) | 0 | 0.127 |
|  | Wild-type | 68 (13) | 90 (26) | 100 (2) |  |
| *CD79A* or *CD79B*^c^ | Mutant in either | 37 (7) | 17 (5) | 0 | 0.176 |
|  | Wild-type for both | 63 (12) | 83 (24) | 100 (2) |  |
| *MYD88* | Mutant | 32 (6) | 10 (3) | 0 | 0.127 |
|  | Wild-type | 68 (13) | 90 (26) | 100 (2) |  |
| *CARD11* | Mutant | 5 (1) | 17 (5) | 0 | 0.380 |
|  | Wild-type | 95 (18) | 83 (24) | 100 (2) |  |
| *NFKBIA* | Mutant | 0 | 17 (5) | 0 | 0.142 |
|  | Wild-type | 100 (19) | 83 (24) | 100 (2) |  |
| *TNFAIP3* | Mutant | 32 (6) | 7 (2) | 0 | 0.045 |
|  | Wild-type | 68 (13) | 93 (27) | 100 (2) |  |
| *TP53* | Mutant | 21 (4) | 55 (16) | 0 | 0.035 |
|  | Wild-type | 79 (15) | 45 (13) | 100 (2) |  |
| *MLL2* | Mutant | 53 (10) | 55 (16) | 50 (1) | 1 |
|  | Wild-type | 47 (9) | 45 (13) | 50 (1) |  |
| *PRDM1* | Mutant | 26 (5) | 0 | 0 | 0.007 |
|  | Wild-type | 74 (14) | 100 (29) | 100 (2) |  |
| *CDKN2A* | Mutant | 63 (12) | 17 (5) | 50 (1) | 0.002 |
|  | Wild-type | 37 (7) | 83 (24) | 50 (1) |  |
| *CDKN2B* | Mutant | 42 (8) | 14 (4) | 50 (1) | 0.041 |
|  | Wild-type | 58 (11) | 86 (25) | 50 (1) |  |
| *EZH2* | Mutant | 0 | 28 (8) | 0 | 0.015 |
|  | Wild type | 100 (19) | 72 (21) | 100 (2) |  |

^a^Includes short variants and fusions/rearrangements

^b^Includes short variants, fusions/rearrangements, and amplifications

^c^For the present exploratory analysis, the *CD79B* mutation subgroup includes patients with short-variant mutations plus the one patient (#680020003) identified with a *CD79B* gene amplification

*ABC* activated B-cell like; *DLBCL* diffuse large B-cell lymphoma; *GCB* germinal center B-cell like

**Supplementary Table 4** Exploratory analyses of clinical outcomes by mutational status

| Gene | ORR, % (*n*) | | | DCR, % (*n*) | | | PFS, months | | | |
| --- | --- | --- | --- | --- | --- | --- | --- | --- | --- | --- |
|  | **Mutant** | **Wild-type** | ***P* value** | **Mutant** | **Wild-type** | ***P* value** | **Mutant** | **Wild-type** | **HR** | ***P* value** |
| *BCL2*^a^ | 16.7 (4/24) | 35.0 (7/20) | 0.185 | 50.0 (12/24) | 55.0 (11/20) | 0.771 | 2.2 | 2.6 | 1.08 | 0.898 |
| *BCL6*^a^ | 16.7 (2/12) | 28.1 (9/32) | 0.698 | 50.0 (6/12) | 53.1 (17/32) | 1 | 2.3 | 2.4 | 0.63 | 0.249 |
| *MYC*^b^ | 12.5 (1/8) | 27.8 (10/36) | 0.656 | 62.5 (5/8) | 50.0 (18/36) | 0.701 | 2.6 | 2.2 | 0.69 | 0.525 |
| *MYC* + either *BCL2* or *BCL6*^a^ | 0 (0/6) | 28.9 (11/38) | 0.311 | 50.0 (3/6) | 52.6 (20/38) | 1 | 2.2 | 2.4 | 0.60 | 0.352 |
| *MYD88* | 25.0 (2/8) | 25.0 (9/36) | 1 | 62.5 (5/8) | 50.0 (18/36) | 0.701 | 2.6 | 2.2 | 0.89 | 0.899 |
| *MYD88 +  CD79A/B* | 16.7 (1/6) | 26.3 (10/38) | 1 | 66.7 (4/6) | 50.0 (19/38) | 0.666 | 2.8 | 2.2 | 1.13 | 0.753 |
| *CARD11* | 14.3 (1/7) | 27.0 (10/37) | 0.659 | 28.6 (2/7) | 56.8 (21/37) | 0.232 | 1.7 | 2.8 | 1.24 | 0.695 |
| *NFKBIA* | 0 (0/3) | 26.8 (11/41) | 0.561 | 0 (0/3) | 56.1 (23/41) | 0.100 | 1.5 | 2.8 | 0.21 | 0.013 |
| *TNFAIP3* | 57.1 (4/7) | 18.9 (7/37) | 0.054 | 57.1 (4/7) | 51.4 (19/37) | 1 | 2.9 | 2.4 | 1.03 | 0.867 |
| *TP53* | 11.8 (2/17) | 33.3 (9/27) | 0.158 | 47.1 (8/17) | 55.6 (15/27) | 0.758 | 1.7 | 2.9 | 0.86 | 0.649 |

^a^Mainly fusions and rearrangements, but short variants also included

^b^Includes fusions, rearrangements, amplifications, and short variants

*DCR* disease control rate; *HR* hazard ratio; *ORR* objective response rate; *PFS* progression-free survival

**Supplementary Fig. 1** Kaplan–Meier curves. **a** Median overall DoR and **b** Median PFS (PPS)

**
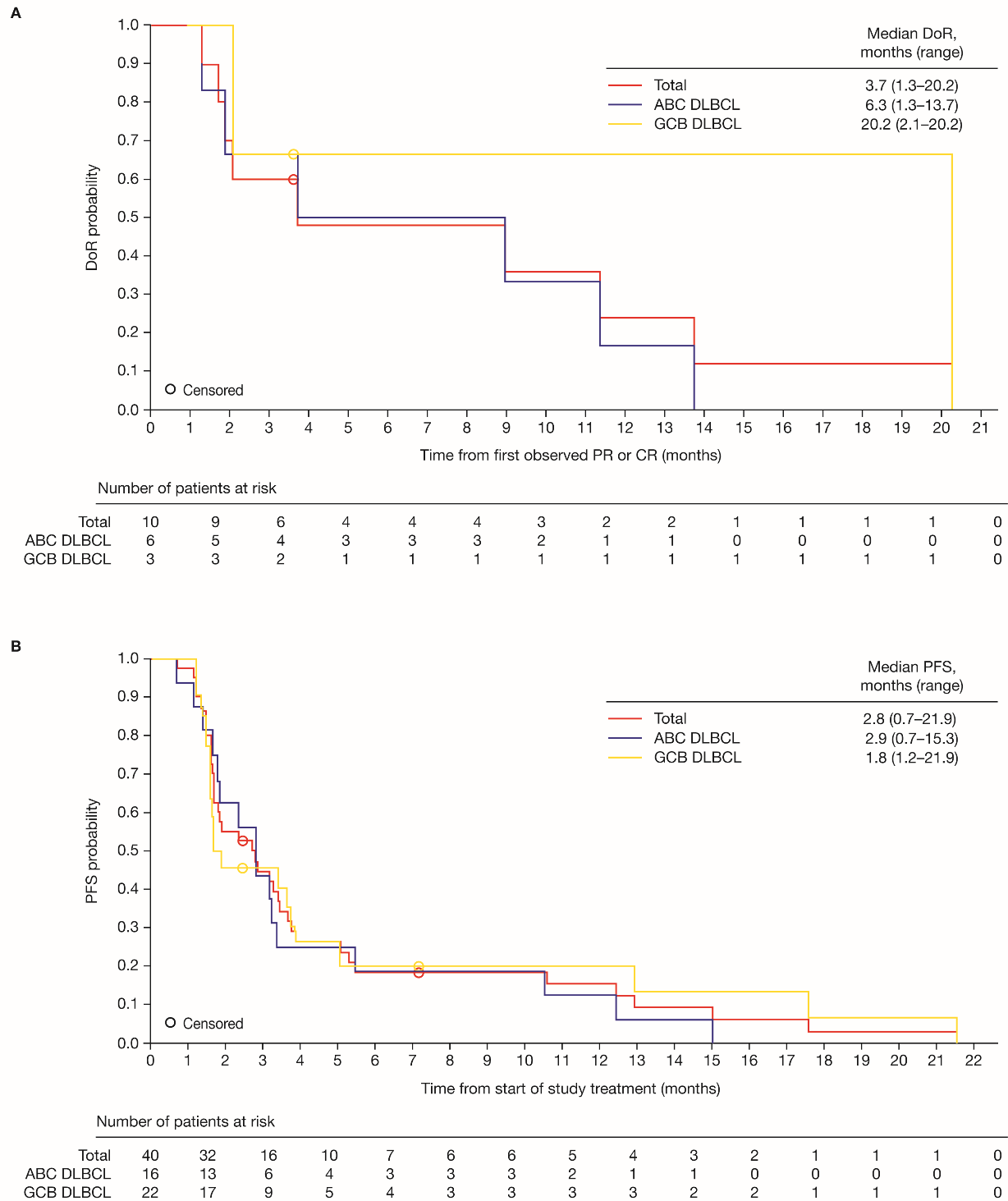
**

*ABC* activated B-cell like; *CR* complete response; *DLBCL* diffuse large B-cell lymphoma; *DoR* duration of response; *GCB* germinal center B-cell like; *PFS* progression-free survival; *PPS* per-protocol set; *PR* partial response

**Supplementary Fig. 2** Genes with ≥2.5 × higher aberration prevalence in one or the other COO subtype (ABC/GCB) (overall cohort)


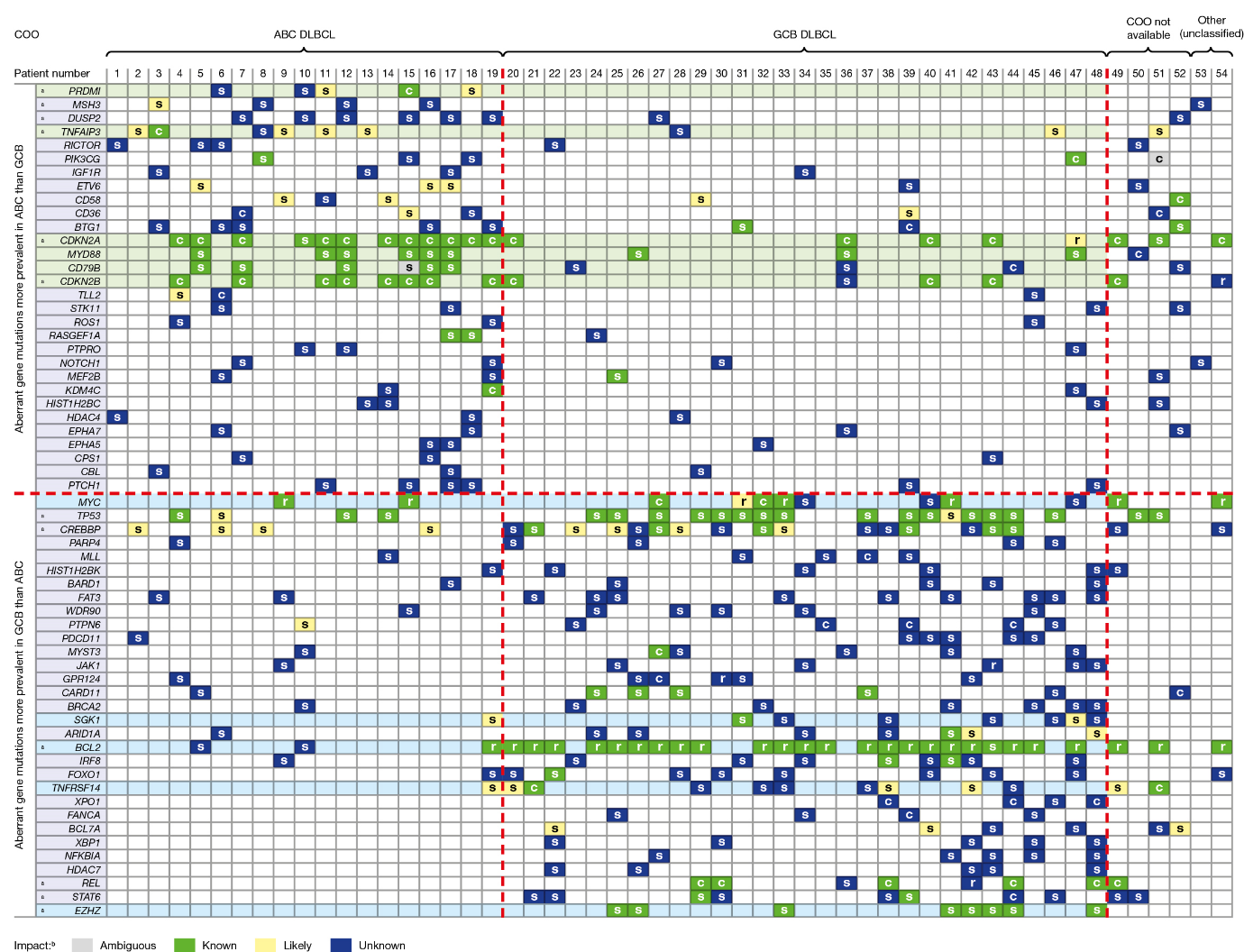


Genes highlighted in green and blue are known to have a higher aberrant prevalence in ABC DLBCL and GCB DLBCL, respectively

^a^Unadjusted *P* < 0.05

^b^Impact of the genetic aberration on clinical outcome as determined by investigator assessment

*ABC* activated B-cell like; *c* copy-number alteration; *COO* cell of origin; *DLBCL* diffuse large B-cell lymphoma; *GCB* germinal center B-cell like; *r* rearrangement; *s* short variant
